# Are asthma and allergy associated with increased root resorption following orthodontic treatment? A meta-analysis

**DOI:** 10.1371/journal.pone.0285309

**Published:** 2023-05-04

**Authors:** Reem Kais Al-Saqi, Athanasios E. Athanasiou, Miltiadis A. Makrygiannakis, Eleftherios G. Kaklamanos

**Affiliations:** 1 Hamdan Bin Mohammed College of Dental Medicine, Mohammed Bin Rashid University of Medicine and Health Sciences, Dubai, United Arab Emirates; 2 Department of Dentistry, European University Cyprus, Nicosia, Cyprus; 3 Department of Orthodontics, School of Dentistry, National and Kapodistrian University of Athens, Athens, Greece; 4 School of Dentistry, Faculty of Health Sciences, Aristotle University of Thessaloniki, Thessaloniki, Greece; University of Zurich, SWITZERLAND

## Abstract

**Objective:**

The aim of this study is to systematically investigate the available evidence from human studies regarding the association of asthma and/or allergy with EARR.

**Materials and methods:**

Unrestricted searches in 6 databases and manual searching were performed up to May 2022. We looked for data on EARR after orthodontic treatment in patients with/without asthma or allergy. Relevant data were extracted, and the risk of bias was assessed. An exploratory synthesis was carried out using the random effects model, and the overall quality of the evidence was assessed with the Grades of Recommendation, Assessment, Development, and Evaluation.

**Results:**

From the initially retrieved records, nine studies met the inclusion criteria (three cohort and six case-control). Overall, increased EARR was observed in the individuals with allergies in their medical history (Standardised Mean Difference [SMD]: 0.42, 95% Confidence Interval [CI]: 0.19 to 0.64). No difference in EARR development was observed among individuals with or without a medical history of asthma (SMD: 0.20, 95% CI: -0.06 to 0.46). The quality of available evidence, excluding studies at high risk, was rated as moderate for the exposure to allergy, and low for the exposure to asthma.

**Conclusion:**

Increased EARR was noted in individuals with allergies compared to the control group, while no difference was observed for individuals with asthma. Until more data become available, good practice would suggest that it is important to identify patients with asthma or allergy and consider the possible implications.

## Introduction

Orthodontic tooth movement takes place by means of application of mechanical forces, of varying degrees of direction, magnitude and duration [1]. Inflammatory-like processes arise from these forces, leading to significant morphological changes in the periodontal structures through bone resorption and apposition [2, 3]. The above pathways involve interactions among cells and the extracellular matrix which are modulated by a variety of systemic hormones, growth factors and cytokines. Numerous studies have shown that osteoblasts and osteoclasts are the main cells controlling orthodontic tooth movement [4].

The main goal of orthodontic treatment is to achieve a long-lasting, aesthetic, healthy and functional occlusion [5]. Despite the benefits of an orthodontic treatment, sometimes, pathologic side-effects may occur as well. One of these is external apical root resorption (EARR) which is a result of the noxious clastic activity of odontoclasts which leads to the loss of root structures [6]. Odontoclasts share many characteristics in terms of morphology and function with osteoclasts [7] and, similarly, develop from the differentiation and specialization of the cells residing in the periodontal ligament (PDL), as well as mononuclear precursor cells from the bone marrow [8].

EARR following orthodontic treatment is considered common and, to some extent, inevitable [9, 10]. Root remodelling is considered a constant feature of orthodontic tooth movement and, as such, EARR should be considered as neither pathologic nor physiologic, but as an unpredictable yet usually clinically acceptable irreversible loss of apical root material [5]. However, when excessive, conservation and functional prognosis of teeth might be affected [11].

Although the exact biological and mechanical parameters responsible for exacerbating resorption of the root tissues during orthodontic treatment or inhibiting their repair remain, to a considerable extent, undetermined, a variety of treatment- and patient-associated factors have been suggested to affect the severity and extent of EARR [12]. Such factors include the duration of orthodontic treatment, the type of appliances, the type and magnitude of forces employed, root morphology and dental trauma, along with systemic supplements, medications, and hormones [13–17].

Asthma and allergic diseases constitute major public health concerns, affecting over 350 million people worldwide, including children and adults [18]. Due to the significant increase in their incidence and prevalence over the last few decades, and the associated socio-economic burden, these diseases have recently gained attention [18, 19]. Moreover, it has been suggested that asthma and allergic diseases may be involved in the exacerbation of EARR associated with orthodontic tooth movement [20, 21]. The immunologic reaction of chronically allergic and asthmatic patients is characterized by systemic imbalances, resulting in an upregulation in the production of inflammatory mediators [22, 23]. These biomolecules might enter the circulatory system, reach the areas of orthodontically induced bone remodelling, and influence cellular recruitment, differentiation and response, and finally, the processes pertinent to tooth movement and EARR development [20, 21]. A recent systematic review concluded that individuals with allergy or asthma do not have a predisposition to EARR [24]. However, not all relevant information was captured, nor was quantitative data synthesis attempted in order to increase power and precision, as well as to explore the settlement of the existing controversies in the evidence.

### Objectives

The objective of the present review was to systematically investigate, quantitively synthesise and critically appraise the quality of the available evidence from human studies regarding the association of asthma and/or allergy with EARR.

## Materials and methods

### Protocol and registration

The present review was based on a protocol developed, registered, carried out and reported following relevant methodological guidelines and registered in Open Science Framework (OSF) (https://osf.io/ftb7z/) [25–30]. As the present study is a systematic review, ethical approval was not required.

### Eligibility criteria

The Population, Exposure, Comparator and Outcomes domains were used to describe the eligibility criteria (S1 Table) [31]. We looked for observational studies evaluating EARR development in individuals with or without a medical history of asthma or/and allergy, after the completion of a full course of comprehensive orthodontic treatment with fixed appliances. We included patients of any age, gender and racial background, but excluded studies that did not include a comparison to a control group. Studies that assessed patients with clefts, syndromes and congenital anomalies of the craniofacial region, as well as patients at the end of an initial phase of treatment with removable and/or fixed appliances were also excluded. Finally, we did not consider animal, in vitro, ex-vivo or in silico studies, as well as reviews, systematic reviews and meta-analyses.

### Information sources and search strategy

One author (XX) developed the detailed search strategies for each of the databases that were searched until May 2^nd^ 2022 (Medline [PubMed], CENTRAL [Cochrane Library; includes records from Embase, CINAHL, ClinicalTrials.gov, WHO’s ICTRP, KoreaMed, Cochrane Review Groups’ Specialized Registers, and records identified by handsearching], Cochrane Database of Systematic Reviews [Cochrane Library], Scopus, Web of Knowledge [including Web of Science Core Collection, KCI Korean Journal Database, Russian Science Citation Index, SciELO Citation Index and Zoological Record] and ProQuest Dissertation and Theses [ProQuest]) (S2 Table). We did not impose any restrictions on the language or date of publication. Duplicates were removed using EndNote’s duplicate identification strategy (EndNote X9™, Clarivate™, Philadelphia, PA, USA) and then manually by XX. We also manually searched the reference lists in relevant articles to identify additional studies (XX and XX).

### Selection process, data collection process and data items

Two authors (XX and XX) assessed the retrieved records for inclusion independently. They were not blinded to the identity of the authors, their institution, or the results of the research. If the abstract was unclear, the full paper was accessed to determine the eligibility for inclusion.

From the finally eligible studies, the following information was extracted in predetermined forms when available: bibliographic details of the study; study design and eligibility; study setting and country, inclusion and exclusion criteria; population characteristics (numbers of study participants; age; gender), characteristics of the orthodontic treatment (e.g. type of appliances (bracket type, slot size, arch wire sequence); treatment duration; treatment with/without extractions, expansion, headgear etc.); type of exposure (allergy; asthma; allergy and asthma); outcome assessment (teeth measured; methods of measurement; unit of analysis; power calculations, group comparability; intrarater and interrater reliability); numerical results, and information regarding the risk of bias assessment domains. Where possible, the characteristics of the population under study and the numerical results were extracted separately for the exposed (RR) and unexposed (non-RR) group (in the case of a cohort study) or for the groups of cases and controls (in the case of a case-control study). The datasets were categorized on the basis of the following medical history characteristics: exposure to allergy, exposure to asthma, exposure to allergy and asthma. If clarifications were needed regarding the published data, or additional material was required, then attempts to contact the corresponding authors through email were made.

### Study risk of bias assessment

The risk of bias in individual studies was assessed by XX and XX independently with the tool for the assessment of risk of bias in observational studies of adverse effects associated with orthodontic treatment [32]. The risk of bias is investigated regarding 4 domains: patient selection; orthodontic treatment; identification of root resorption; and analysis of outcomes [32]. Studies that presented high risk of bias in Domains 2, 3, and/or 4 were assigned an overall “high” risk of bias rating. Assessments were subsequently entered into the Risk-of-bias VISualization (robvis) web application [33]. In all the aforementioned processes, disagreements were settled by discussion with XX; following the relevant suggestions, kappa statistics were not calculated [27].

### Effect measures and synthesis methods

Data on EARR external root resorption presented some variety, including continuous data from linear measurements and ordinal information from various grading scales, and were combined according to Higgins et al. [27]. In order for the summary measures to be comparable across studies, the differences in EARR between the exposed and non-exposed groups were expressed using the standardised mean difference (SMD) (together with 95% confidence interval—CI) [27, 34, 35].

Given the methodological differences among the included studies (Tables 1 and 2), a meta-analytical synthesis was performed on an exploratorary basis only. For this purpose, the random effects method for meta-analysis was used to combine data [27]. The magnitude of the summary effect size was interpreted according to Sawilowsky [36]. In order to facilitate the appreciation of the difference in EARR between the exposed and non-exposed groups, summary SMDs were re-expressed into difference in the percentage of EARR, based on the information from the Nanekrungsan et al. [37] study and odds ratios [27]. Ordinal scales were dichotomized according to the severity of EARR (mild vs. moderate/severe) and summarized using methods for continuous data [27]. Due to the lack of an adequate number of retrieved studies, it was not possible to calculate the corresponding 95% prediction intervals [27].

**Table 1:** General characteristics.

Study	Inclusion and exclusion criteria	Analyzed sample ^a^	
**McNab et al. 1999 [46]** Cohort study University of Queensland Australia	**Inclusion criteria:** medical history updated at the beginning of Tx; complete records, Tx plan and history **Exclusion criteria:** patients with dental agenesis, invaginations and taurodontism. ***Exposed group*:** unmedicated/medicated asthma patients. ***Non-exposed group*:** no medical conditions, matched for age and gender (minimum 2 controls/asthmatic).	**Exposed group [asthma]:** 44; 517 apices Age: 14.2 ±2.3y Tx duration: 1.8 ±0.4y % males: 41.9; % extractions: 56.8	**Non-exposed group:** 97; 1159 apices Age: 13.9 ±1.8y Tx duration: 1.9 ±0.5 y % males: 39.2; % extractions: 59.8
**Shim & Davidovitch 2003 [47]** Case-control study Kyung Hee University South Korea	**Inclusion criteria:** Complete records, Tx plan and history. ***EARR group*:** ≥1 tooth with 25% ERR. ***Control group*:** matched for age, gender, Tx duration, type of malocclusion	**EARR group:** 51 patients Age: 16.1 ±3.3y Tx duration: 2.13 ±0.73y % males: 41.2; % extractions: 64.7	**Control group:** 51 patients Age: 15.4 ±4.1y Tx duration: 2.31 ±0.7y % males: 41.2; % extractions: 39.2
**Aghili et al. 2006 [48]** Case-control study Private practice Iran	**Inclusion criteria:** Complete records, Tx plan and history **Exclusion criteria:** patients with oligodontia, dilaceration, invagination; root resorption because of impacted tooth. ***EARR group***: ≥1 root/tooth with EARR ≥ grade 2, no EARR before Tx. ^b^ ***Control group*:** matched for age, gender, ANB, use of headgear and Tx duration	**EARR group**: 70 patients Age: 14.9 ±3.5y Tx duration: 2 ±0.4y % males: 35.7; % extractions: 64.3	**Control group:** 70 patients Age: 15.8 ±2.6y Tx duration: 2 ±0.6y % males: 35.7; % extractions: 71.4
**Nishioka et al. 2006 [49]** Case-control study Kyushu University Japan	**Inclusion criteria:** Complete records, Tx plan and history. ***EARR group*:** ≥1 root/tooth with 25% ERR. ***Control group*:** matched for age, sex, Tx duration and type of malocclusion	**EARR group:** 60 patients Age: 16.8 ±5.9y Tx duration: 3.1 ±1.19y % males: 30; % extractions: 73.3	**Control group:** 60 patients Age: 17.7 ±5.9y Tx duration: 2.96 ±0.56y % males: 30; % extractions: 61.6
**Nanekrungsan et al. 2012 [37]** Cohort study Chiang Mai University Thailand	**Inclusion criteria:** Complete dental arches, complete records, pre-/post-Tx PA radiographs. **Exclusion criteria:** Crown fracture of abrasion during Tx, poor radiographs ***Exposed group*:** Patients with allergic condition ***Non-exposed group*:** Patients without allergic condition	**181 patients**	
		**Exposed group [allergy]:** 88 teeth	**Non-exposed group:** 472 teeth
**Ioi et al. 2015 [50]** Case-control study Kyushu University Japan	**Inclusion criteria:** Complete records, Tx plan and history. ***EARR group*:** ≥1 tooth with 25% ERR. ***Control group*:** matched for age, sex, Tx duration and type of malocclusion	**EARR group:** 100 patients Age: 17.8 ±6.2y Tx duration: 3.1 ±1.1y % males: 29	**Control group:** 100 patients Age: 18.1 ±5.2y Tx duration: 2.9 ±0.8y % males: 29
**Malan 2017 [51]** Cohort study Loma Linda University USA	**Inclusion criteria**: Pre-/post-Tx CBCT scans with the same machine **Exclusion criteria:** Missing or not fully formed M CI; phase 1 Tx; Mx surgical cases; changes in incisal contour of CI. ***Exposed group*:** Asthma patients. ***Non-exposed group*:** Non-asthmatic patients.	**291 patients:** 120M, 71F; Age: 17 ±9.5 y; Tx duration: 2.22 ±0.7y	
		**Exposed group [asthma]:** 66 teeth	**Non-exposed group:** 516 teeth
**de Melo et al. 2018 [52]** Case-control study Ingá Dental School Brazil	**Inclusion criteria:** Complete records, Tx plan and history; good pre-/post-Tx PA radiographs of the Mx and Md Is. ***EARR group***: EARR ≥ grade 3.^c^ ***Control group***: EARR ≥ grade 0–2. ^c^	**EARR group:** 69 patients Age: 15.09 ±3.44y Tx duration: 2.72 ±1.07 years % males: 46.3; % extractions: 59.4	**Control group:** 614 patients Age: 14.37 ±2.76 years Tx duration: 2 ±0.6 years % males: 48.8; % extractions: 38.59
**Pastro et al. 2018 [53]** Case-control study Uningá University Center Brazil	**Inclusion criteria:** Complete records, Tx plan and history; good pre-/post-Tx PA radiographs of the Mx and Md Is. ***EARR group***: EARR ≥ grade 3. ^c^ ***Control group***: EARR ≥ grade 0–2. ^c^	**EARR group**: 93 patients Age: 14.57 ±2.97 years Tx duration: 2.41 ±0.99y % males: 43; % extractions: 62.3	**Control group**: 507 patients Age: 14.21 ±2.45y Tx duration: 1.81 ±0.83y % males: 50.09; % extractions: 37.8

^a^ Age at the beginning of treatment and treatment duration are reported in means and standard deviations.

^b^ Sharpe, W., Reed, B., Subtelny, J.D., and Polson, A. (1987) Orthodontic relapse, apical root resorption, and crestal alveolar bone levels. *American Journal of Orthodontics and Dentofacial Orthopedics*, 91, 252–258.

^c^ Levander, E., and Malmgren, O. (1988). Evaluation of the risk of root resorption during orthodontic treatment: a study of upper incisors. *European Journal of Orthodontics*, 10, 30–38.

ERR: external root resorption; CI(s): central incisor(s); I(s): Incisor(s); M: males: F: females; Tx: treatment; Md: mandibular; Mx: maxillary; Nm: not mentioned; Pm: premolars; OPG: orthopantomogram; PA: periapical; RR: root resorption; CEJ: cementoenamel junction; y: years

**Table 2:** Intervention and outcome measurement characteristics.

Study	Intervention characteristics	Teeth assessed and EARR assessment methods	Additional information
**McNab et al. 1999 [46]** Cohort study University of Queensland Australia	Fixed appliances: EW/non-EW Tx plan: +/- extractions, HG	All teeth except Mx & Mn incisors ^a^ OPG [EARR classification based a grading scale] ^b^ Unit of analysis: the tooth apex	Power calculations: Yes, retrospective Group comparability: Yes [age, gender, Tx duration, %EW, %HG, OJ, OB, %non-extraction] Reliability: Kappa score
**Shim & Davidovitch 2003 [47]** Case-control study Kyung Hee University South Korea	Fixed appliances: Tx plan: +/- extractions, orthognathic surgery	All teeth OPG [Measurement of root length] Unit of analysis: the individual	Power calculations: Nm Group comparability: Yes [age, gender, Tx duration, type of malocclusion; need for orthognathic surgery] Reliability: Νm
**Aghili et al. 2006 [48]** Case-control study Private practice Iran	Fixed appliances: 0.018” EW Tx plan: +/- extractions	All teeth except Mn incisors OPG [EARR classification based a grading scale] ^b^^,^^c^ Unit of analysis: the individual	Power calculations: Nm Group comparability: Yes [age, gender, Tx duration, ANB, use of HG] Reliability: Nm
**Nishioka et al. 2006 [49]** Case-control study Kyushu University Japan	Fixed appliances: EW Tx plan: +/- extractions	All teeth OPG [Root length (CEJ to apex)] ^d^ Unit of analysis: the individual	Power calculations: Nm Group comparability: Yes [age, gender, Tx duration, type of malocclusion] Reliability: ANOVA
**Nanekrungsan et al. 2012 [37]** Cohort study Chiang Mai University Thailand	Fixed appliances: standard/preadjusted EW Tx plan: +/- extractions	Mx incisors PA [% of ERR] ^e^	Power calculations: Nm Group comparability: Nm Reliability: Pearson’s product-moment correlation
**Ioi et al. 2015 [50]** Case-control study Kyushu University Japan	Fixed appliances: Tx plan: +/- extractions	All teeth OPG [Measurement of root length] ^f^ Unit of analysis: the individual	Power calculations: Nm Group comparability: Yes [age, gender, Tx duration, type of malocclusion] Reliability: Nm
**Malan 2017 [51]** Cohort study Loma Linda University USA	Fixed appliances: Tx plan: +/- extractions, expansion	Mx central incisors CBCT [Difference in pre- to post-Tx tooth length] ^g^ Unit of analysis: the tooth	Power calculations: Nm Group comparability: Nm Reliability: Intra-class correlation coefficient
**de Melo et al. 2018 [52]** Case-control study Ingá Dental School Brazil	Fixed appliances: preadjusted EW Tx plan: +/- extractions	Md and Mx incisors PA [EARR classification based a grading scale] ^h^ Unit of analysis: the individual	Power calculations: Yes [based on a pilot study] Group comparability: Yes [gender, type of malocclusion, +/- extractions] Reliability: Kappa score
**Pastro et al. 2018 [53]** Case-control study Uningá University Center Brazil	Fixed appliances: Tx plan: +/- extractions	Mx and Md central incisors PA [EARR classification based a grading scale] ^i^^,^^j^ Unit of analysis: the individual	Power calculations: Nm Group comparability: Yes [age, gender, type of malocclusion, +/- extractions] Reliability: Kappa score

^a^ Excluded: palatal root of the upper first molars, teeth with incomplete root formation, teeth with apices which could not be visualized accurately, teeth with pre-Tx resorption.

^b^ Sharpe, W., Reed, B., Subtelny, J. D., & Polson, A. (1987) Orthodontic relapse, apical root resorption, and crestal alveolar bone levels. *American Journal of Orthodontics and Dentofacial Orthopedics*, 91, 252–258.

^C^ Roots/teeth with EARR ≥ grade 2 were considered to have moderate/severe ERR.

^d^ Excluded: teeth with incomplete root formation, teeth with apices which could not be visualized accurately, teeth whose crown lengths were obviously different before and after Tx because of image distortion.

^e^ Root length from CEJ to apex; measurements corrected for enlargement.

^f^ CEJ to apex.

^g^ Incisal edge to apex.

^h^ Levander, E., and Malmgren, O. (1988). Evaluation of the risk of root resorption during orthodontic treatment: a study of upper incisors. *European Journal of Orthodontics*, 10, 30–38.

^i^ Malmgren, O., Goldson, L., Hill, C., Orwin, A., Petrini, L., and Lundberg, M. (1982) Root resorption after orthodontic treatment of traumatized teeth. *American Journal of Orthodontics*, 82, 487–491.

^j^ The highest degree of degree of resorption of all teeth was considered. Teeth with endodontic treatment or tooth reimplantation were excluded.

CBCT: Cone beam computed tomography; CEJ: cementoenamel junction; ERR: external root resorption; EW: Edgewise; HG: headgear; Md: mandibular; Nm: not mentioned; Mx: maxillary; OB: overbite; OJ: overjet; OPG: orthopantomogram; PA: periapical radiograph; Tx: treatment; +/-: with/without.

To identify the presence and the extent of heterogeneity between studies, the overlap of 95% confidence interval for the results of individual studies was inspected graphically, and the I^2^ statistic was calculated [27]. All analyses were carried out with Comprehensive Meta-Analysis Software version 3 (©2014 Biostat Inc., Englewood, New Jersey, USA). Significance (a) was set at 0.05, except for 0.10 used for Q tests [38].

### Certainty assessment and additional analyses

As per protocol, analyses were to be carried out for “small-study effects” and publication bias but were not finally performed due to the lack of an adequate number of studies [27]. For the same reason, subgroup analyses considering the effect of ethnic background and the use of headgear on orthodontically induced EARR were not performed [27]. Where possible, we used meta-regression to explore through univariate regression models, whether the results were modified by the mean age at the beginning of treatment, the mean treatment duration, the percentage of male participants and the percentage of extractions in the studied population. Sensitivity analyses comparing the results between studies having the tooth, the apex or the patient as a unit of measurement and analyses excluding the studies at high risk of bias were conducted. Finally, despite the lack of extensive information, the quality of available evidence regarding the differences in EARR between the exposed and the non-exposed groups of orthodontic patients, excluding studies at high risk of bias, was assessed in order to adopt a structured and transparent approach in formulating an interpretation of the evidence [30, 39]. As cohort and case-control studies may be a suitable study design for adverse outcomes [27], the assessment started from the grade “high” [30, 40].

## Results

### Study selection

**Fig 1:**
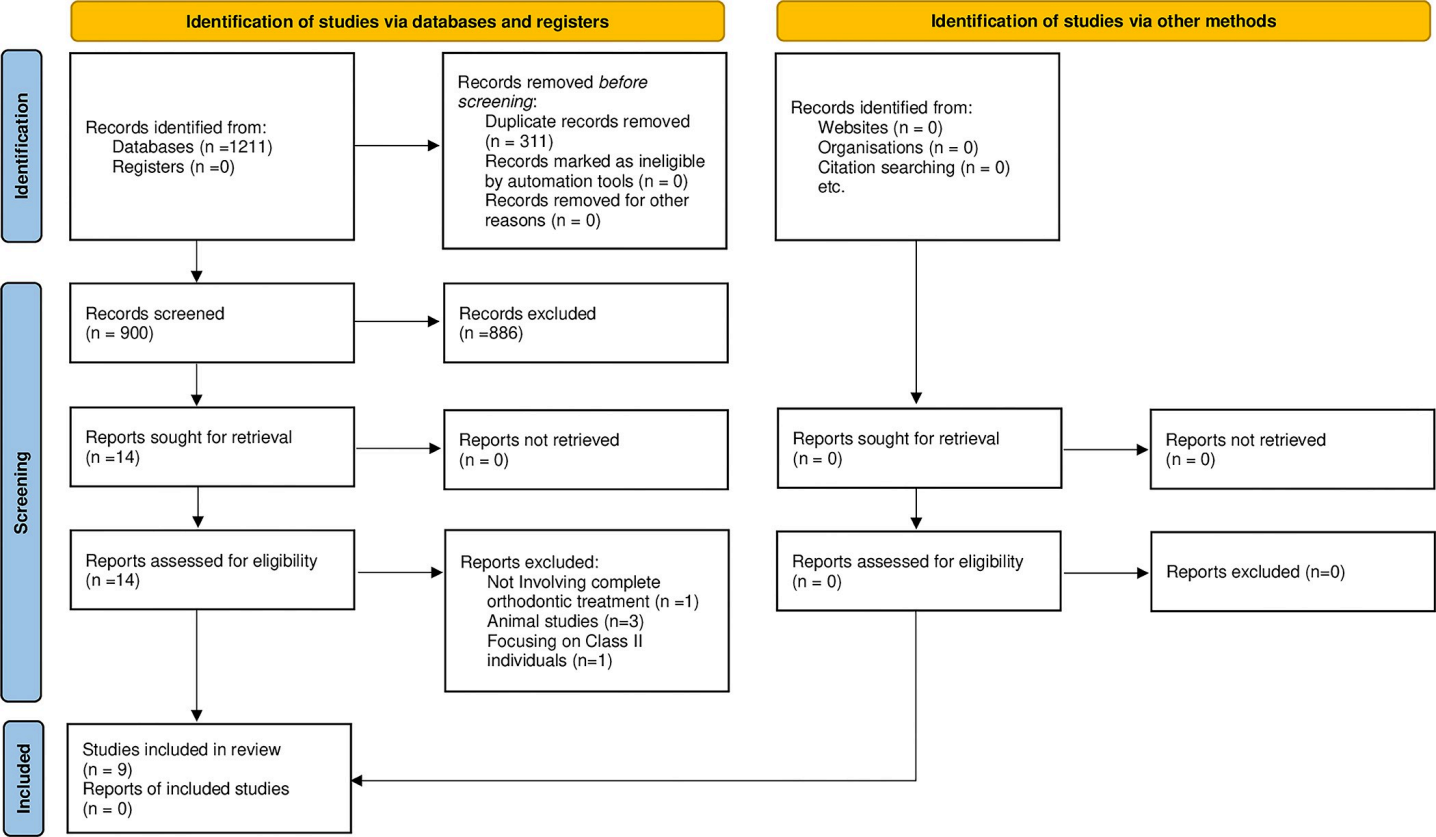
PRISMA statement 2020 flow diagram.

Following database searching, we collected 1211 records. 311 records were identified as duplicates, and a further 886 were excluded on the basis of their title and abstract. Subsequently, 14 papers were assessed for eligibility and 5 of them were excluded. The reasons of exclusion were the following: one study involved individuals that had not undergone complete orthodontic treatment [41], another one focused exclusively on Class II patients [42], while the other three were conducted on experimental animals [43–45]. Finally, 9 papers were included in the review (Fig 1) [37, 46–53].

### Study characteristics

The retrieved eligible studies were published between 1999 and 2018, had been conducted mostly in University clinics, and assessed EARR development before and after comprehensive orthodontic treatment with fixed appliances in 2458 patients. Three of these papers had a cohort design [37, 46, 51], while the rest of them were case-control studies [47–50, 52, 53] (Tables 1 and 2).

Information regarding the individual’s medical history was obtained from their treatment files. The association of allergy with EARR development was investigated by Shim and Davidovitch [47], Aghili et al. [48], Nishioka et al. [49], Nanekrungsan et al. [37] and Pastro et al. [53]. The association with asthma was studied by McNab et al. [46], Shim and Davidovitch [47], Aghili et al. [48], Nishioka et al. [49], Malan [51] and de Melo et al. [52]. Shim and Davidovitch [47] and Ioi et al. [50] assessed also the association of both allergy and asthma present in the medical history of the patients.

Five studies used orthopantomograms (OPG) for the assessment of root resorption [46–50], three used periapical radiographs [37, 52, 53] and one used cone beam computed tomography (CBCT) [51]. The assessment included all teeth [47, 49, 50] or excluded only the mandibular [48] or all incisors [46]. Malan [51] focused on maxillary centrals, Nanekrunngsan et al. [37] on maxillary centrals and laterals, and finally, de Melo et al. [52] and Pastro et al. [53] assessed all incisors.

Four of the included studies used grading scales for the assessment of resorption, assigning different resorption grades to the investigated teeth [Sharpe et al. [54] scale [46, 48]; Malmgren et al. [55] scale [52, 53]], whereas the rest measured the actual root or tooth length and used the measurements to identify an excessive resorption group [47, 49, 50], calculated the percentage of root resorption [37] or the pre- to post-treatment length differences [51].

The duration of the orthodontic treatment varied between the retrieved studies, and some of them explicitly mentioned that preadjusted edgewise brackets were used in all or a part of the investigated patients [37, 46, 48, 49, 52]. All studies included patients who had extractions as part of their treatment plan. Malan [51] included patients who had maxillary expansion, and Shim and Davidovitch [47] included patients who had undergone orthognathic surgery as well. Seven studies demonstrated comparability between the investigated groups for various characteristics, usually including age, gender, treatment durations and the inclusion or not of extractions in the treatment plan.

Only de Melo et al. [52] mentioned that they had performed sample size calculation based on a pilot study, while McNab et al. [46] did the calculations retrospectively. Most studies reported on intrarater/interrater reliability, with the exception of Shim and Davidovitch [47], Aghili et al. [48] and Ioi et al. [50].

### Risk of bias in studies

**Fig 2:**
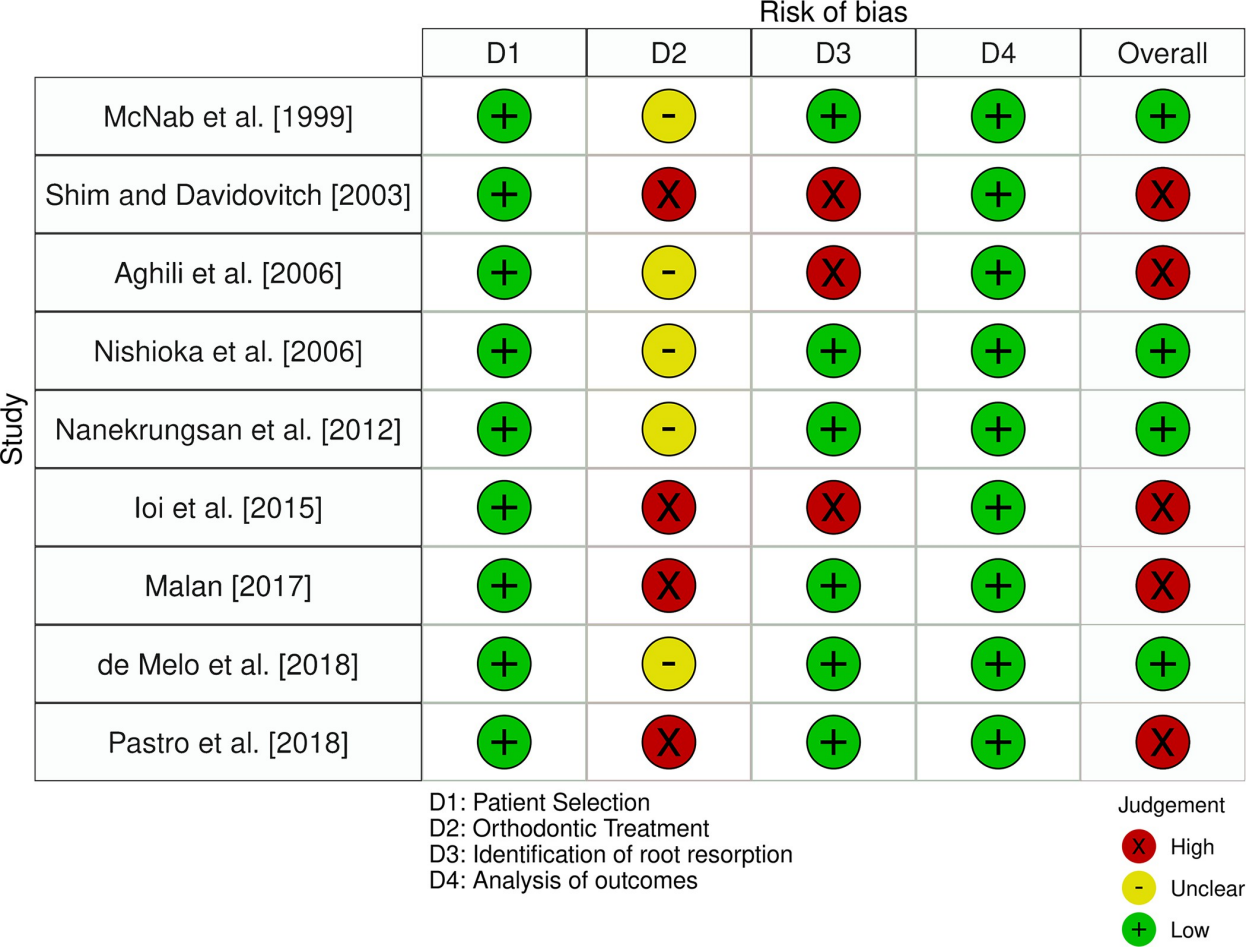
Risk of bias assessment.

The risk of bias assessment with the tool for observational studies of adverse effects associated with orthodontic treatment [32] is presented in Fig 2. Overall, five studies were considered to exhibit high risk of bias [47, 48, 50, 51, 53]. In terms of the “patient selection” item, all studies received an assessment of low risk. For the “orthodontic treatment” domain, four studies did not present a description of the actual treatment provided and were rated at high risk [47, 50, 51, 53], whereas in the rest reporting was inadequate and were rated at unclear risk. Regarding the risk of bias in the “identification of root resorption” the majority of studies were assessed at low risk, except for three of them, assessed at a high risk of bias due to lack of assessment of intrarater/interrater reliability [47, 48, 50]. Finally, the risk of bias in regard to “analysis of outcomes” was low.

### Association of asthma and/or allergy with orthodontically induced EARR

**Fig 3:**
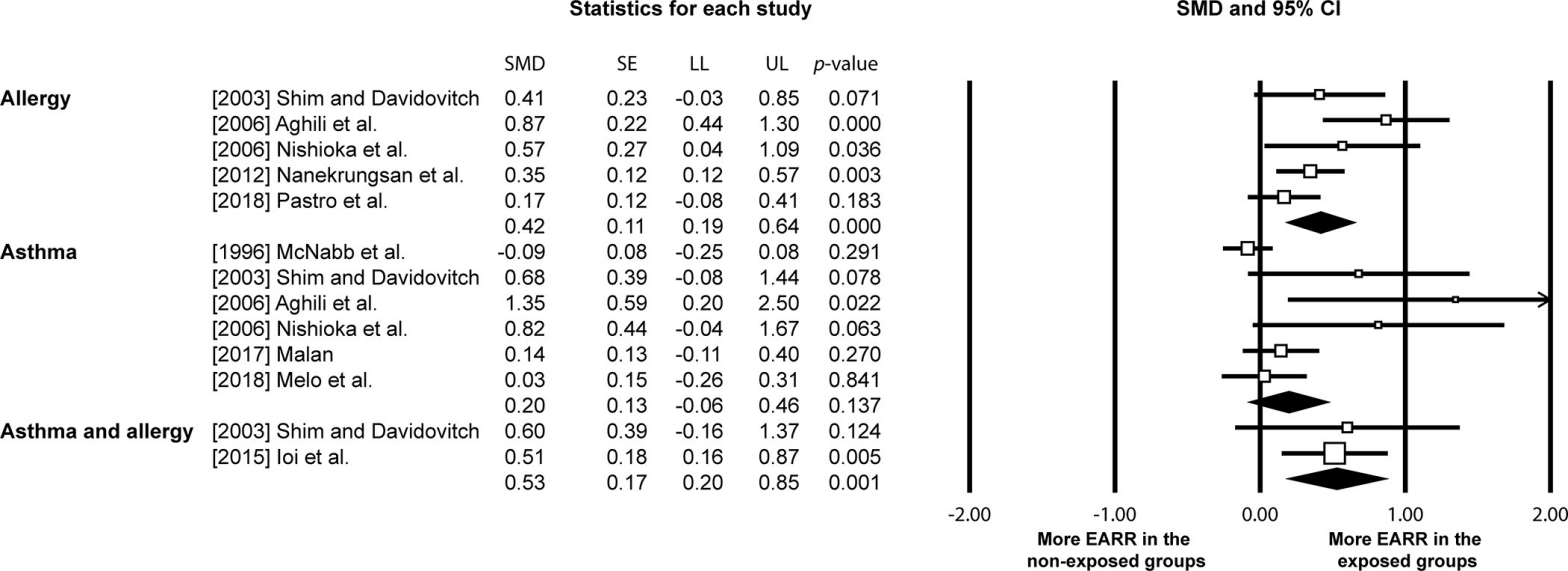
Effect of exposure to allergy and/or asthma to external apical root resorption (EARR) development following orthodontic treatment [CI: confidence interval; LL: lower limit; SE: standard error; SMD: standardised mean difference; UL: upper limit].

**Fig 4:**
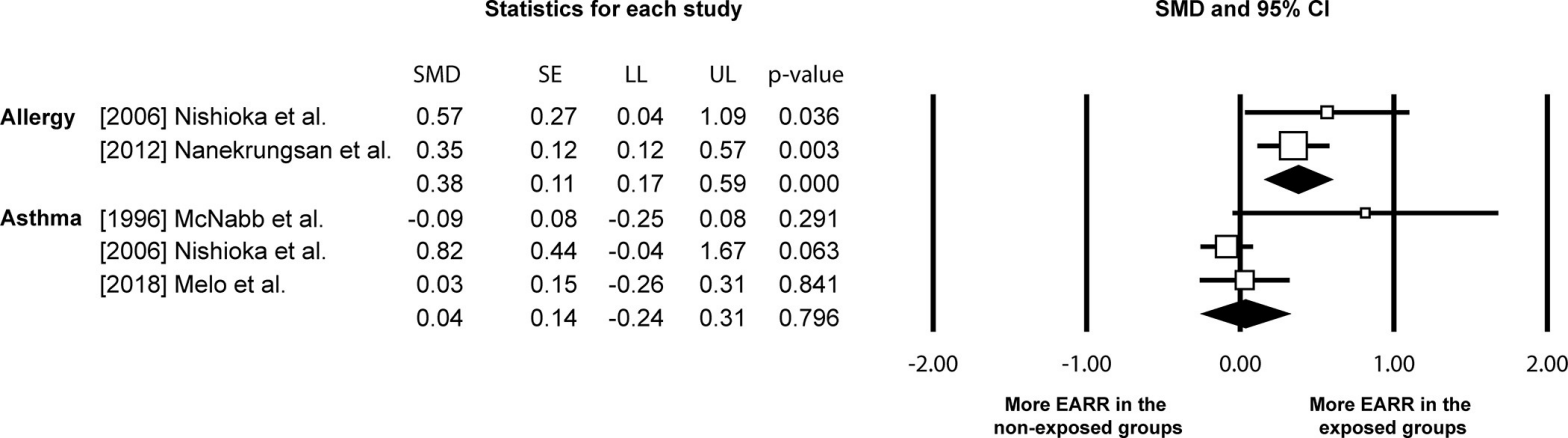
Effect of exposure to allergy and/or asthma to external apical root resorption (EARR) development following orthodontic treatment, excluding studies at high risk [CI: confidence interval; LL: lower limit; SE: standard error; SMD: standardised mean difference; UL: upper limit].

Overall, more EARR was noted in the individuals with just allergy or both allergy and asthma in their medical history (allergy: SMD: 0.42, 95% CI: 0.19 to 0.64, p = 0.000, I^2^: 52.76%) (allergy and asthma: SMD: 0.53, 95% CI: 0.20 to 0.85, p = 0.001, I^2^: 0%) with the SMDs corresponding to medium effect sizes (Fig 3). The OR for experiencing greater EARR in the groups with medical history of allergy only and both allergy and asthma was 1.94 (95% CI: 1.50–2.50) and 2.61 (95% CI: 1.45–4.71). Respectively, that corresponded to 4% and 5% more EARR. No difference in EARR development was observed between individuals with/without medical history of asthma alone (asthma: SMD: 0.20, 95% CI: -0.06 to 0.46, p = 0.137, I^2^: 63.50%), with the effect size being small, the OR calculated to be 1.07 (95% CI: 0.86–1.33) and the difference in EARR between the groups of 0.3%. Quantitative data synthesis excluding studies at high risk of bias produced corroborating results (Fig 4) (S3 Table).

The series of exploratory meta-regressions that included the intercept and mean age at the beginning of treatment, the mean treatment duration, the percentage of male participants and the percentage of extractions in the studied population as predictors, did not show any statistically significant effect on EARR development in post-orthodontic patients with allergy or asthma compared to the non-exposed groups (Table 3). Sensitivity analyses comparing the results between studies with various unit of measurements showed that the results were robust (S4 Table). Regarding the differences in EARR between orthodontic patients exposed to asthma or allergy alone, compared to those non-exposed [30, 39], the quality of available evidence was rated as moderate for the exposure to allergy and low for the exposure to asthma (S3 Table).

**Table 3:** Series of meta-regressions for the effect of various parameters on EARR development in post-orthodontic patients.

					Test of the model^1^			Goodness of fit			
	Coefficient	SE	LL	UL	Q	df	p-value	Q	df	p-value	R^2^ analogue
**Individuals with medical history of allergy vs. non-exposed patients**											
**Age at the beginning of Tx**	0.12	0.16	-0.19	0.43	0.61	1	0.433	5.05	2	0.080	0.04
**Tx duration**	0.14	0.45	-0.74	1.02	0.09	1	0.758	7.27	2	0.026	0.00
**% male participants**	-0.02	0.01	-0.04	0.01	2.19	1	0.139	4.87	3	0.181	0.33
**% extractions**	0.01	0.01	-0.01	0.03	1.44	1	0.230	5.60	3	0.132	0.00
**Individuals with medical history of asthma vs. non-exposed patients**											
**Age at the beginning of Tx**	0.11	0.10	-0.09	0.31	1.08	1	0.298	10.09	4	0.039	0.00
**Tx duration**	-0.01	0.02	-0.04	0.03	0.11	1	0.745	13.08	4	0.010	0.00
**% male participants**	-0.04	0.02	-0.08	0.01	2.14	1	0.143	12.42	4	0.014	0.00
**% extractions**	0.01	0.01	-0.01	0.03	1.17	1	0.279	13.66	4	0.008	0.00

^1^ Random effects (Method of Moments), Z-Distribution

## Discussion

### Summary of evidence

Even though EARR is a common side effect of orthodontic treatment, noticeable amounts of resorption during a relatively short period, such as the course of orthodontic treatment, is not so common [9, 10]. Nonetheless, it has been suggested that individuals who have comorbidities, such as allergy and asthma, that disturb the immune system, might be at a risk of developing severe root resorption [20, 21]. Based on the information provided in the included studies, more EARR was noted in the individuals with allergy compared to the control group, while no difference was observed in patients with asthma. The GRADE assessment was moderate for allergy and low for asthma, implying that the true effect is probably close to the estimate for allergy, and potentially markedly different for asthma, providing insights to the relevant recommendations. Good practice would be to focus on the identification of patients with allergy or asthma, and to take the possible implications into consideration.

Only nine studies were found to investigate the association of allergy and asthma with EARR after comprehensive orthodontic treatment. The consequent lack of extensive information is rather unexpected, considering the prevalence of the diseases and that severe EARR might not only complicate the orthodontic treatment plan and biomechanics, but also teeth conservation and their functional prognosis [11]. Owman-Moll and Kurol [41] also investigated in, a case-control design, the association of allergy as a predisposing factor for root resorption development. Medical information was obtained through patient interviews, and a differentiation was made for the self-reported cases and those verified by consultation with the physician or by on-going medication. However, in the aforementioned study, the intervention did not include a complete course of comprehensive orthodontic treatment, thus curtailing generalizability. After buccal movement of maxillary premolars for 1 to 7 weeks, the experimental teeth were extracted and examined histologically. Individuals with allergy showed an increased risk for root resorption, but this risk was not statistically significant.

The results from animal experiments regarding the effect of allergen sensitization on orthodontically induced EARR have suggested that it may increase the susceptibility to root resorption. A histomorphometric study of the PDL of Wistar rats in the initial period of tooth movement showed an enhanced response to mechanical stimuli in the animals sensitized nasally with ovalbumin. The PDL was more compressed at the pressure side and more stretched in the tension side, a response that could potentially indicate increased bone turnover, as well as osteoclastogenesis and odontoclastogenesis [43]. Murata et al. [44] investigated, in rats sensitized with ovalbumin, the amount of orthodontic tooth movement and root resorption, and compared it to that measured in control animals. They observed that both amounts of movement and root resorption were more pronounced in the former group. Moreover, the levels of RANKL and proinflammatory cytokines were upregulated in the PDL of the sensitized animals. At the same time, an increase in leukotriene B4 (LTB4), a potent lipid mediator of allergic inflammation, and enzymes of the 5-lipoxygenase pathway, the biosynthetic pathway of leukotrienes, was observed. Moreover, low doses of aspirin suppressed root resorption in allergen-sensitized rats, as well as the expressions of RANKL, proinflammatory cytokines, and LTB4. On the contrary, Aghili et al. [45] did not observe a statistically significant difference in root resorption development between sensitized and non-sensitized animals.

EARR following orthodontic tooth movement is considered to be a complicated phenomenon, associated with a multitude of parameters and inflammatory related pathways [13]. The various implicated factors are believed to be both biological [56] and mechanical [57–60] and might not be involved in the same degree in all affected individuals, leading to different patterns of EARR in terms of severity and extent. Thus, sometimes no single explanation can be given, nor are we able to unequivocally predict individual susceptibility [13].

Even though the located information was not extensive, some points arising from these data relevant to the treatment of patients with allergy and/or asthma might be considered. Based on the GRADE assessment, the observed association between allergy and EARR is probably close to the true effect from the exposure. In contrast, no effect from the exposure to asthma was noted, but, potentially, the estimation could differ markedly in the future as new information becomes available. Until more research results are accessible, it could be considered safe practice to identify patients with allergy and/or asthma and consider any pertinent adjustment to the treatment plan and mechanotherapy. In these cases, application of lower forces, a lower frequency of appointments, radiographic follow-up, as well as paying attention to the other factors that have been associated with root resorption development might be warranted [11, 61]. Such mechanical or treatment related factors include tooth movement into the labial or cortical bone [62], long treatment duration and increased magnitude of force [63–66], the amount of apical displacement [67], the inclusion of extractions in the treatment plan [15] and the use of inter-maxillary elastics [58]. Moreover, biological or patient-related factors should be considered. Some studies have reported that orthodontically induced EARR is more prevalent in older individuals [68, 69], although others have not observed similar associations [15]. Dental anomalies (ectopia, agenesis, taurodontism, etc.) [70, 71], teeth with blunt, dilacerated or narrow roots [57, 70–72], individuals with parafunctional oral habits [73, 74], traumatised teeth [55, 75], as well as the effect of the medication that individuals use for asthma and allergic diseases [76] or other medical conditions [16, 17] all warrant our attention.

### Strengths and limitations

The adherence to widely accepted methodological standards counts as a strength for the current review. The searches were unrestricted and comprehensive, all processes were duplicated, and discrepancies were settled by discussion. The existing limitations arise mainly from the nature or the characteristics of the studies and the information retrieved.

As already mentioned, the located studies were limited, rendering quantitative assessments indicative and exploratory until additional research becomes available. Nevertheless, alternative summaries can be less transparent and potentially less valid [77], and even information from two studies can be synthesized as long as pooling is meaningful [78]. Moreover, quantitative data synthesis increases power and precision, while also allowing for the settlement of controversies in the evidence. At this point, it should be clarified that given the methodological differences among the included studies, it was conducted on an exploratory basis. An important source of potential heterogeneity in effect estimates for adverse effects is variation in outcome definition and measurement [27]. In the context of the present review, a potential limitation might stem from the way the exposure to the investigated risk factor, (i.e., allergy or asthma) was ascertained. The patients’ medical history from their orthodontic treatment file was used without further verification, clarification or elaboration on the exact nature and severity of the condition and the associated symptoms or the medication used. Moreover, the studies that investigated the association of allergy and root resorption development did not specify whether they were referring to airway allergy or another kind of allergic condition. However, it was not possible to obtain further information from the respective authors.

Other variables that were investigated in the included studies were systemic diseases, medications use, type of initial malocclusion, treatment plan, duration of treatment, gender, age, ethnicity, root morphology, overjet, overbite, history of trauma, parafunctional habits, mouth breathing, periodontal problems and pre-treatment root resorption. However, these parameters were not always considered as confounding factors in the analysis of the association of exposure to asthma or allergy and EARR development.

Furthermore, the reported results might have been affected by the methodology employed for the assessment of resorption. Although orthodontically-induced EARR involves a three-dimensional process, most studies assessed it using conventional radiographs, orthopantomograms or periapical radiographs and thus, limiting the investigation to two dimensions. In these cases, root resorption can only be discovered on the apex, as well as the mesial and distal surfaces. Additionally, both methods are considered as inadequate tools for accurate measurement and unsuitable for the identification of the progression of the relative minor root resorption that might occur during the course of treatment with fixed orthodontic appliances [79]. The standardized procedure of taking intra- or extra-oral radiographs is technique sensitive and might cause distortion to the tooth image, thus creating inherent limitations in the measurement reliability, especially for the panoramic radiographs, predominantly in the incisor region [53]. These expected errors associated with the two-dimensional radiographic techniques might be avoided by the use of cone-beam computed tomography [80].Five of the included studies made actual root or tooth length measurements [34, 47, 49, 50, 51], whereas the rest assigned a score based on ordinal grading scales, a fact that could potentially have influenced the precision of the results. Moreover, all studies assessed multiple teeth without employing relevant statistical adjustments. Although Pastro et al. [53] considered only the highest degree of resorption from the assessed teeth, an approach that dealt with the problem of repeated observations, at the same time, this analysis obscured the extent and the severity of the phenomenon in the other incisors, potentially ignoring important information.

### Recommendations for future research

Although pronounced root resorption over a relatively short period, such as the course of orthodontic treatment, is not to be expected [9, 10], in order to investigate the phenomenon more comprehensively, and, considering the prevalence of allergy and asthma [18], further research is warranted. Since it is not expected to that randomized studies will be conducted in order to investigate this subject, it is sensible to suggest conducting well-controlled observational studies [29, 30]. Particular importance should be placed on possible ways to control such confounding factors as outlined previously, as well as bias in the measurement of outcomes. In this respect, the use of cone-beam computed tomography is advisable [61, 80]. Moreover, stable and easily located reference points should be considered. For example, regarding the cementoenamel junction, although anatomically stable, it is harder to replicate its exact location in comparison to the incisal edge [51].

## Conclusions

More EARR was noted in individuals with allergy compared to the control group, while no difference was observed for individuals with asthma. Until more data become available, good practice would suggest that it is important to identify patients with asthma or allergy and consider the possible implications.

## Supporting information

S1 Checklist PRISMA 2020 checklist. (DOCX)

S1 Table Eligibility criteria. (DOCX)

S2 Table Strategy for database search (up to May 2^nd^, 2022). (DOCX)

S3 Table Quality of available evidence [excluding studies at high risk of bias]. (DOCX)

S4 Table Sensitivity analyses. (DOCX)
