# Supplementary material for: Are asthma and allergy associated with increased root resorption following orthodontic treatment? A meta-analysis
Source: PLoS One. 2023 May 4;18(5):e0285309. doi: 10.1371/journal.pone.0285309 (PMC10159203; doi:10.1371/journal.pone.0285309)
Supplement: S1 Table — (DOCX) [file pone.0285309.s002.docx]

**S1 table.** Eligibility criteria.

| **Domain** | **Inclusion criteria** | **Exclusion criteria** |
| --- | --- | --- |
| **Participants** | • Orthodontic patients of any age, gender and racial background, having undergone a full course comprehensive orthodontic Tx with fixed appliances of any type regarding technique, bracket characteristics, performance or not of extractions, etc. | • Patients at the end of an initial phase of Tx with removable and/or fixed appliances only (e.g., fixed or removable expanders only, fixed or removable functional appliances only, 2x4 or 2x6 appliances, etc.).  • Studies focusing on a specific type of malocclusion.  • Patients with clefts, syndromes or congenital anomalies of the craniofacial region. |
| **Exposure(s)** | • Medical history of asthma and/or allergy. |  |
| **Comparisons** | • Individuals without medical history of asthma and/or allergy |  |
| **Outcomes** | • Measurements on the extent and/or the severity of external apical root resorption along with measurements of dispersion (assessed using linear measurements or grading scales), based on X-rays (orthopantomograms, periapical radiographs, CBCT, etc.). | • Not quantified measurements and qualitative assessments; absence of measures of central tendency and dispersion. |

Tx: treatment
