# Supplementary material for: Are asthma and allergy associated with increased root resorption following orthodontic treatment? A meta-analysis
Source: PLoS One. 2023 May 4;18(5):e0285309. doi: 10.1371/journal.pone.0285309 (PMC10159203; doi:10.1371/journal.pone.0285309)
Supplement: S2 Table — (DOCX) [file pone.0285309.s003.docx]

**S2 Table.** Strategy for database search (up to May 2^nd^, 2022).

| **Database** | **Search strategy** | **Hits** |
| --- | --- | --- |
| **Medline via PubMed** | (orthodon* OR “orthodontic force” OR “mechanical force”) AND ("tooth movement" OR “orthodontic movement” OR “orthodontic anchorage” OR “root resorption”) AND (allergy OR allergic* OR sensitiv* OR hypersensitiv* OR ovalbumin OR OVA OR “Dust Mite” OR HDM OR ascaris OR aspergill* OR “cotton dust” OR latex OR DRA OR cockroach OR asthma* OR airway) | **253** |
| **Cochrane Central Register of Controlled Trials** | (orthodon* OR “orthodontic force” OR “mechanical force”) AND ("tooth movement" OR “orthodontic movement” OR “orthodontic anchorage” OR “root resorption”) AND (allergy OR allergic* OR sensitiv* OR hypersensitiv* OR ovalbumin OR OVA OR “Dust Mite” OR HDM OR ascaris OR aspergill* OR “cotton dust” OR latex OR DRA OR cockroach OR asthma* OR airway) in Title, Abstract, Keywords in Trials - (Word variations have been searched) | **31** |
| **Cochrane Database of Systematic Reviews** | (orthodon* OR “orthodontic force” OR “mechanical force”) AND ("tooth movement" OR “orthodontic movement” OR “orthodontic anchorage” OR “root resorption”) AND (allergy OR allergic* OR sensitiv* OR hypersensitiv* OR ovalbumin OR OVA OR “Dust Mite” OR HDM OR ascaris OR aspergill* OR “cotton dust” OR latex OR DRA OR cockroach OR asthma* OR airway) in Title, Abstract, Keywords in Trials - (Word variations have been searched) | **1** |
| **Scopus** | TITLE-ABS-KEY (orthodon* OR (orthodontic force) OR (mechanical force)) AND ((tooth movement) OR (orthodontic movement) OR (orthodontic anchorage) OR (root resorption)) AND (allergy OR allergic* OR sensitiv* OR hypersensitiv* OR ovalbumin OR OVA OR (Dust Mite) OR HDM OR ascaris OR aspergill* OR (cotton dust) OR latex OR DRA OR cockroach OR asthma* OR airway)) | **613** |
| **Web of Science™** | TOPIC: ((orthodon* OR “orthodontic force” OR “mechanical force”) AND ("tooth movement" OR “orthodontic movement” OR “orthodontic anchorage” OR “root resorption”) AND (allerg* OR sensitiv* OR hypersensitiv* OR ovalbumin OR OVA OR “Dust Mite” OR HDM OR ascaris OR aspergill* OR “cotton dust” OR latex OR DRA OR cockroach OR asthma* OR airway))  Timespan: All years. Databases: WOS, KJD, RSCI, SCIELO, ZOOREC.  Search language=Auto | **301** |
| **ProQuest Dissertations and Theses Global** | ti((orthodon* OR “orthodontic force” OR “mechanical force”) AND ("tooth movement" OR “orthodontic movement” OR “orthodontic anchorage” OR “root resorption”) AND (allergy OR allergic* OR sensitiv* OR hypersensitiv* OR ovalbumin OR OVA OR “Dust Mite” OR HDM OR ascaris OR aspergill* OR “cotton dust” OR latex OR DRA OR cockroach OR asthma* OR airway)) OR ab((orthodon* OR “orthodontic force” OR “mechanical force”) AND ("tooth movement" OR “orthodontic movement” OR “orthodontic anchorage” OR “root resorption”) AND (allergy OR allergic* OR sensitiv* OR hypersensitiv* OR ovalbumin OR OVA OR “Dust Mite” OR HDM OR ascaris OR aspergill* OR “cotton dust” OR latex OR DRA OR cockroach OR asthma* OR airway)) [Full text] | **7** |
