# Supplementary material for: Are asthma and allergy associated with increased root resorption following orthodontic treatment? A meta-analysis
Source: PLoS One. 2023 May 4;18(5):e0285309. doi: 10.1371/journal.pone.0285309 (PMC10159203; doi:10.1371/journal.pone.0285309)
Supplement: S3 Table — (DOCX) [file pone.0285309.s004.docx]

**S3 Table.** Quality of available evidence [excluding studies at high risk of bias].

| - **Quality assessment** | | | | | | - **Effect size** | - **Quality** | |
| --- | --- | --- | --- | --- | --- | --- | --- | --- |
| - **Studies** | - **Risk of bias** | - **Inconsistency** | - **Indirectness** | - **Imprecision** | - **Other** | - **MD and 95% CI** |  | |
| **Differences in EARR between orthodontic patients exposed to allergy alone *vs.* non-exposed** | | | | | | | | |
| 2 | - Not serious | - Not serious^1^ | - Not serious | - Serious^2^ | - None | - 3.72% more EARR in the EG group [from 1.66 to 5.79; p=0.000] | - ⨁⨁⨁◯ | **MODERATE** |
| - **Differences in EARR between orthodontic patients exposed to asthma alone *vs.* non-exposed** | | | | | | | | |
| 3 | - Not serious | - Serious^3^ | - Not serious | - Serious^2^ | - None | - 0.39% more EARR in the EG group [from -2.35 to 3.94; p=0.796] | - ⨁⨁◯◯ | - **LOW** |

- CI: Confidence Interval; EARR: external root resorption; EG: exposed group; MD: Mean Difference

^1^I-squared was 0%; ^2^The result was based on a small number of patients. ^3^I-squared may represent moderate heterogeneity.
