# Supplementary material for: Are asthma and allergy associated with increased root resorption following orthodontic treatment? A meta-analysis
Source: PLoS One. 2023 May 4;18(5):e0285309. doi: 10.1371/journal.pone.0285309 (PMC10159203; doi:10.1371/journal.pone.0285309)
Supplement: S4 Table — (DOCX) [file pone.0285309.s005.docx]

**S4 Table.** Sensitivity analyses.

|  | **Test of the model^1^** | | |
| --- | --- | --- | --- |
|  | **Q** | **df** | **p-value** |
| **Allergy** | 0.14 | 1 | 0.712 |
| **Asthma** | 1.78 | 1 | 0.182 |

^1^ Random effects (Method of Moments), Z-Distribution
